# Clear Cell Sarcoma (Malignant Melanoma) of Soft Parts: A Clinicopathologic Study of 52 Cases

**DOI:** 10.1155/2012/984096

**Published:** 2012-05-30

**Authors:** O. Hocar, A. Le Cesne, S. Berissi, P. Terrier, S. Bonvalot, D. Vanel, A. Auperin, C. Le Pechoux, B. Bui, J. M. Coindre, C. Robert

**Affiliations:** ^1^Melanoma Committee, Gustave Roussy Institute, 114 Rue Edouard Vaillant, 94805 Villejuif, Cedex, France; ^2^Sarcoma Committee, Gustave Roussy Institute, 114 Rue Edouard Vaillant, 94805 Villejuif, Cedex, France; ^3^Department Of Radiology, Gustave Roussy Institute, 114 Rue Edouard Vaillant, 94805 Villejuif, Cedex, France; ^4^Department Of Biostatistics And Epidemiology, Gustave Roussy Institute, 114 Rue Edouard Vaillant, 94805 Villejuif, Cedex, France; ^5^Sarcoma Group Of Bergonié Institute, 229 Cours De L'argonne, 33076 Bordeaux, Cedex, France

## Abstract

Clear cell sarcomas are aggressive, rare soft tissue tumors and their classification among melanoma or sarcoma is still undetermined due to their clinical, pathologic, and molecular properties found in both types of tumors. This is a retrospective study of 52 patients with CCS seen between April 1979 and April 2005 in two institutions. The EWS-ATF-1 fusion transcript was studied in 31 patients and an activating mutation of the *BRAF* or *NRAS* gene was researched in 22 patients. 30 men and 22 women, with a mean age of 33 were studied. Forty-three tumors (82.69%) were located in the extremities, specially the foot (19 tumors). Median initial tumor size was 4.8 cm (1 to 15 cm). Necrosis involving more than 50% of the tumor cells was found in 14 cases (26.92%). High mitotic rate (>10) was found in 25 cases (48.07%). The EWS/ATF-1 translocation was found in 28 (53.84%) of 31 patients studied, and mutation of *BRAF* or *NRAS* was found in only 2 of 22 patients analyzed cases (3.84%). Among the tumor-associated parameters, only tumor size (>4 cm) emerged as a significant prognostic factor. Forty-nine patients had a localized disease at diagnosis (94.23%) and underwent surgical resection immediately (90%) or after neoadjuvant chemotherapy (CT) (10%). Various CT regimens were used in 37 patients (71.15%) with no significant efficacy. The 5- and 10-year OS rates were 59% and 41%, respectively. Tumor size was the only emerging prognosis factor in our series. Complete surgical resection remains the optimal treatment for this aggressive chemoresistant tumor.

## 1. Introduction

Clear cell sarcoma (CCS) is a rare malignant tumor that was described first by Enzinger in 1965 [1]. CCS shows a predilection for the deep soft tissues of the lower extremities close to the tendon, fascia, or aponeuroses [2]. It occurs preferentially in adolescents and young adults and is associated with a high propensity of local recurrence, regional lymph node metastases, and distant metastases [1, 2]. Because of its close clinic and histologic kinship with malignant melanoma (high frequency of lymph node metastases, presence of melanin, ultrastructural evidence of melanosomes, and immunohistochemical staining for S-100 protein and melanoma-associated antigen HMB-45), Chung and Enzinger proposed the name malignant melanoma of soft parts [2]. However, despite these similarities, CCS and melanoma should be considered 2 distinct entities. Unlike melanomas, most CCS tumors are characterized by a recurrent chromosomal translocation, t(12; 22), resulting in fusion of the EWS gene on 22q12 with the ATF1 gene on 12q13 [3]. Several fusion transcript types have been described, with a predominance of type 1 fusing exon 8 of EWS with exon 4 of ATF1 and type 2 fusing exon 7 of EWS with exon 5 of ATF1 [4]. The prevalence of t(12; 22) fusion transcripts detected by molecular techniques has been reported in only 3 small series of 10 patients, 12 patients, and 9 patient with CCS [3–5].

High incidence of activating mutations in the BRAF gene has been reported in melanoma cell lines, melanoma short-term cultures, primary and metastatic melanomas, and nevi [6]. All the mutations were detected in the kinase domain of the BRAF gene and found in exons 11 and 15. The most common mutation (V599E) is the T1796A single-base substitution in exon 15, leading to an exchange of valine for glutamic acid at position 599 [6].

This mutation was not reported in soft tissue sarcomas including a small series of 8 CCS [4]. Compared with the extensive literature regarding the histopathologic nature of the disease, very little is known about the clinical and molecular features of CCS. The main obstacle to gaining a thorough understanding of the clinical and molecular behavior of CCS is the rarity of the disease.

We describe the clinicopathological data of 52 patients with clear cell sarcoma with emphasis on prognostic factors and treatment outcomes. Mutational status on BRAF, NRAS gene, and cytogenetic study of translocation (12, 22) (q13, q12) were also studied (Table 1).

## 2. Patients and Methods

Between April 1979 and April 2005, 52 consecutive cases of CCS were registered at the Gustave Roussy, Villejuif (46 patients) and Bergonie, Bordeaux (6 patients) institutes.

Complete information on clinical data, treatment modalities, and outcome was reviewed in all cases to exclude an antecedent or a concurrent diagnosis of primary cutaneous melanoma. When the initial biopsies were done outside the institutes, the histopathologic diagnosis was confirmed by Gustave Roussy or Bergonie Institute expert of pathologists. Histologic criteria for the diagnosis of CCS are the intimate association of small solid aggregates of round-to-fusiform, pale-staining cells within the dense connective tissue of tendons and aponeuroses, the fine reticular stroma surrounding the cells, the relative uniformity of cells showing abundant clear cytoplasm, and scattered multinucleated giant cells. Mitotic figures are generally infrequent. A confirmation of diagnosis is obtained by immunohistochemistry with a diffuse or focal positivity for S-100 protein and melanocyte-associated antigen HMB-45, positivity for neuron-specific enolase (NSE). Thirty-one cases were examined for the presence of EWS/ATF1 transcripts by real-time PCR on paraffin-embedded tissues and frozen samples.

DNA was prepared from paraffin-embedded tissues of 22 CCS (16 cases from GRI, 6 cases from BI) carrying the t(12; 22) translocation. PCR amplification of BRAF exons 11 and 15 as well as NRAS codons 12, 13, and 61 was performed, and PCR products were sequenced.

The following demographic and treatment factors were examined for prognostic importance: patient age (≤30 years, >30 years), sex, tumor size (≤4 cm, >4 cm), location, symptoms, clinical staging, microscopic surgical margin, chemotherapy and radiotherapy, mitotic rate (>2 per 10 high power fields after examining and averaging data from 20 HPF), and presence of necrosis.

With respect to tumor location, proximal sites included the trunk and proximal extremities (upper arm and thigh), and distal sites included distal extremities (below or at the elbow and below or at the knee joint). The microscopic surgical margin was determined histologically on the resected specimens and was classified as negative (no tumor cells at the inked margin) or positive (tumor cells at the inked margin). Patients received multimodality treatment, including surgery, radiotherapy, and chemotherapy. Wide excision of the primary tumor with a negative surgical margin was attempted whenever possible. If necessary, amputation/disarticulation was planned to achieve a negative surgical margin.

All time-to-event endpoints were computed by using the kaplan-Meier method [7].

Patients who died of causes unrelated to CCS were censored at time of death. Potential prognosis factors were identified by univariate analysis using the log-rank test. Independent prognostic factors were evaluated with Cox proportional-hazards regression using a stepwise selection procedure [8]. To arrive at a parsimonious multivariate model, covariates were selected into the model only if they contributed significantly to the fit of the model. The criterion used to select covariates into the model was the score chi-square statistic. Differences at *P* < .05 were considered significant. Statistical analyses were performed using the StatView 5 statistical package. 

## 3. Results

The study group comprised 30 men (57.69%) and 22 women (42.30%), and patients ranged in age from 6 years to 81 years (median, 33 years). Forty-two percent of patients were aged ≤30 years, and 94% were aged <60 years.

Forty-three tumors (82.69%) were located in the extremities, eight tumors (15.38%) were located in the trunk, and one tumor was located in the scalp. The most common tumor site was the foot (19 tumors), followed by the hand (13 tumors), and the thigh (9 tumors). Fourteen tumors (26.92%) were considered proximal, and 38 tumors (73.07%) were considered distal.

Fifty patients (96.15%) had a mass that had been enlarging slowly for periods ranging from six weeks to four years (median, 15 weeks). Pain and/or tenderness were observed in 30 patients (57.69%), and this was frequently the main reason for seeking medical advice. In two patients, acute urinary retention was the first sign of the disease. No patients had a history of melanoma or unusual pigmented skin lesions.

At presentation the malignant nature of the swelling was seldom suspected, and in thirty-six patients (69.23%), a biopsy was performed. Nevertheless in sixteen patients (30.76%), the lesion was thought to be benign on clinical grounds, and initial clinical diagnosis included epithelial cyst (7 patients) exostose (4 patients), haematoma (2 patients), and neurinoma, lipoma, and carpal tunnel syndrome (1 patient each).

Initial diagnosis was made at another institution in all cases, with incisional or excisional biopsy. All cases were reviewed by the Gustave Roussy or the Bergonie Institute soft tissue pathologists.

The tumors ranged in size from 1 cm to 15 cm (median, 4, 86 cm). The tumors measured <4 cm in 30 patients (57.69%) and between 4 cm and 15 cm in 21 patients (40.38%). Tumor size was unknown in one patient.

In all cases, the tumor cells were arranged in nests, clefts, and an alveolar pattern, separated by fibrous septa. Some dyscohesive cells were seen lying in spaces. The cells showed necrosis less than 50% in 33 patients (63.46%), more than 50% in 14 patients (26.92%), and no evidence of necrosis in 5 patients (09.61%). Of the fourteen patients who had necrosis more than 50%, ten patients died. The number of mitoses was less than 10 per high power fields (10 HPF) in 27 patients (51.92%), between 10 and 20 per 10 HPF in 21 patients (40.38%), and more than 20 per HPF in 4 patients (7.69%). Forty tumors (76.92%) were grade 2, and twelve tumors (23.07%) were grade 3 using the FNCLCC grading system.

HMB45 was diffusely positive in forty-four tumors (84.61%) and focally positive in 8 patients (15.38%). S100 protein was largely positive in forty-four patients (84.61%), focally positive in 5 cases (09.61%), and negative in 3 cases (5.76%). Melan A and MITF were expressed in many cases (76.92% and 84.6% of cases, resp.) including S100 protein-negative tumors. Vimentin could be detected in 3 cases (5.76%). None of our cases had expressions of keratin. Only one of the 22 CCS harboured a BRAF mutation (V599E); it was the well-known valine to glutamic acid change in position 599. No exon 11 mutation were found. One mutation of NRAS (codon 61) was also found with no overlap with the BRAF mutation. The EWS/ATF-1 fusion transcript was detected in 28 patients of the 31 paraffin-embedded CCS, and 3 of the 31 patients were negative. The two tumours that presented a BRAF or NRAS mutation also presented the ATF1-EWS fusion gene and were considered atypical. However, the absence of cutaneous involvement, histologic features (spindle and a few epithelioid cells arranged in a nested pattern), and immunohistochemistry (diffuse positivity for S100 protein and HMB45) strongly suggested a diagnosis of CCS.

Forty-nine patients (94.23%) presented with localized disease; forty-four patients (84.61%) of them underwent surgical resection of primary tumor. Nineteen patients (36.53%) underwent complete surgical resection with negative margins; twenty-five patients (48.07%) had residual microscopic disease and underwent adjuvant therapy especially radiotherapy.

The last five patients with localized disease received neoadjuvant chemotherapy before surgical resection in order to reduce tumors size and make surgical resection possible.

Two patients (3.84%) presented with disease metastatic to lymph nodes. One patient presented with disease metastatic to both lymph nodes and lung.

Definitive surgical management included wide local excisions in 29 patients, local excision in 11 patients, radical compartment resection in 4 patients, and amputation in 3 patients. Adjuvant treatment was given to 42 patients and consisted of radiotherapy in 40 patients (76.92%) with a median of 50 grays. This radiotherapy was associated to adjuvant chemotherapy in 10 patients, and 2 patients received adjuvant chemotherapy alone.

29 patients (55, 76%) developed local recurrence. The time interval from initial treatment to local relapses ranged from 1 month to 20 years, with a median of 38 months. Local recurrence was seen in 55% of the 29 patients at 5 years and 63% at 10 years. Sixteen of these patients received additional surgical treatment and 3 had amputation. Of the other 10 patients (19.23%), they were already known to have regional lymph node metastases and/or had disseminated disease. Of the 29 patients (55, 76%) who had local recurrences, 19 (36.53%) died of the tumors, three (5.76%) are alive with disease, but only seven (13.46%) patients are alive free of disease.

No local recurrences occurred in 23 patients (44.23%) who had surgical resection with negative margins (16 patients) or with positive margins associated to adjuvant radiotherapy (7 patients). The overall survival rate without local recurrence was 30% at 5 years and 16% at 10 years.

Nineteen patients developed regional lymph node metastases at 5 years in 34% and at 10 years in 41% of them. Of these patients, six (11.53%) had lymph node metastases within less than 1 year of the followup, three patients (5.76%) between 1 and 2 years, and 10 patients (19.23%) between 2 and 7 years of the followup. Regional lymph node dissection was performed on fourteen patients and was associated with regional isolated limb perfusion with melphalan and tumor necrosis factor alpha in one case. Five patients had already distant metastases. 

Thirty patients developed distant metastases: 43% of them at 5 years and 62% at 10 years. Of them, twenty-seven patients died because of disease and three are alive with disease (time of followup: 09, 63, 84 months). Distant metastases most frequently involved the lungs (27 patients), bones (2 patients), and distant lymph nodes (1 patient).

Twenty-four patients received some form of chemotherapy, and three patients had surgical treatment (accessible metastases sites and single localisation).

Various chemotherapeutic regimens for musculoskeletal sarcomas or for malignant melanoma, in adjuvant aim or in curative one, were employed, including actinomycin, Adriblastina, cyclophosphamide, carboplatine, cisplatine; dacarbazine, etoposide, fotemustine; iosfamide, interleukin 2, ifosfamide (3 g/m²) + dactinomycin (1,5 mg/m²) + vincristine (1,5 mg/m²); regional isolated limb perfusion with melphalan and tumor necrosis factor alpha, methotrexate, MAID: doxorubicine (15 mg/m²) + dacarbazine (250 mg/m²/j) + ifosfamide (2–2,85 g/m²/j), vincristine; VACA: cyclophosphamide (1,2 g/m²) + doxorubicine (30 mg/m²) + dactinomycin (0,5 mg/m²) + vincristine 1,5 mg/m²; intrferon and taxol.

In twenty-one patients, chemotherapy was discontinued, usually after three courses, because of tumor progression (18 patients) or major hematotoxicity (3 patients). Local radiotherapy for distant metastases sometimes briefly alleviated the pain and was mostly given to patients with skeletal metastases.

**Figure 1:**
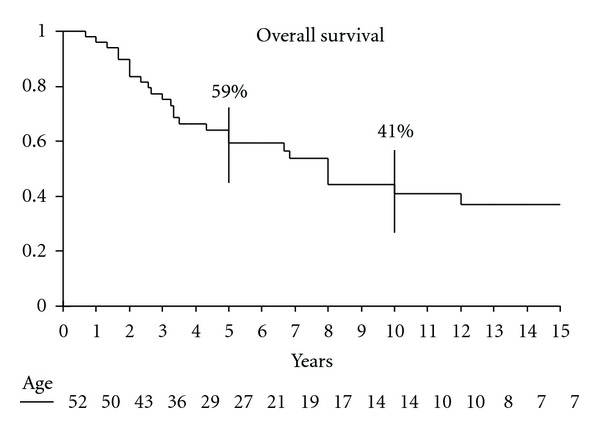
Overall survival of 52 patients with clear cell sarcoma.

**Figure 2:**
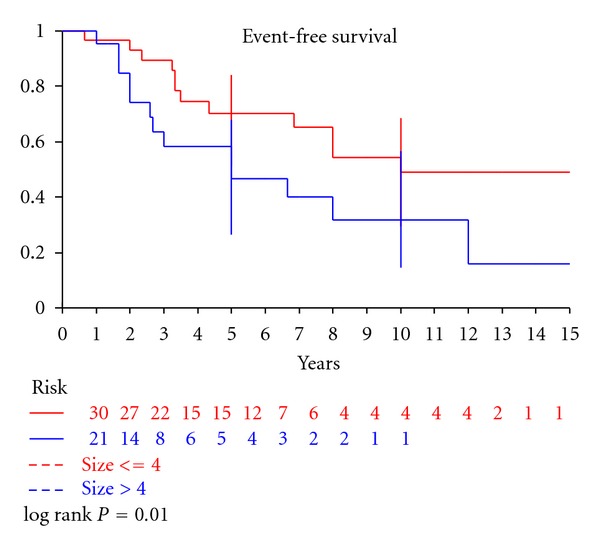
Event-free survival of 52 patients with clear cell sarcoma according to tumor size (*P* = 0.01).

The overall survival rate was 59% at 5 years and 41% at 10 years (Figure 1). Median time of follow up was 120 months (11–348). On multivariate analysis, only tumor size (*P* : 0.01) emerged as a significant prognostic factor (Table 2 and Figure 2). 

## 4. Discussion

These results confirmed the known clinical features of CCS originally reported by Enzinger [1]: arising primarily in young adults, predominance in distal extremities, and presenting as a slowly enlarging mass that has been evident for several months or even years. The principal sites of the neoplasm are the extremities, especially the region of the foot and ankle. The head, neck, and trunk regions are only rarely involved. In our study, forty-three tumors (82.69%) were located in the extremities. The most common tumor site was the foot (19 tumors). Fourteen tumors (26.92%) were considered proximal, and 38 tumors (73.07%) were considered distal. Our findings also revealed several important factors that predict the clinical behavior of CCS and disclosed problems awaiting solution.

Over half of all clear cell sarcoma reported in the literature measured less than 4 cm [2], a finding in accord with our series in which 57.69% of patients had lesions that measured less or equal to 4 cm. CCS mainly afflicts young adults between the ages of 20 to 40 years [4, 5], but in rare instances it may occur in the extremes of ages. In our study, the mean age was 33 and 26 years (range 6–81 years). Although some reports have found predominance in females [6], the gender distribution between males and females is equal [2]. CCS was slightly more common in male than female patients in our series.

CCS is characterized by a recurrent chromosomal translocation t(12; 22), which results in fusion of the EWS gene on 22q with the ATF1 gene on 12q [4]. This genomic abnormality may represent a good marker for identifying these tumors. In the current study, 90.32% of the analyzed tumors (28 of 31 tumors) had this translocation.

As previous studies [4–7], our data further suggests that CCS and conventional melanoma develop through different genetic pathways. Indeed, CCS is a sarcoma subtype, and the characteristic EWS/ATF1 translocation, resulting in the chimeric protein EWS/ATF1, is responsible for the melanocytic differentiation. EWS/ATF1 can bind the transcription factor MITF which in turn can lead to melanocytic differentiation with expression of the protein involved in melanin synthesis: tyrosinase, melanin, HMB45. Because of the extensive clinical, histologic, and immunohistochemical similarities with melanoma, we decided to analyze whether CCS also has mutations in the BRAF and NRAS gene. No mutation of the BRAF nor NRAS gene was identified in 95.54% of the analyzed tumors (21 of 22 tumors). The two tumours which were found to harbor mutations in the BRAF and NRAS gene also presented the ATF1-EWS fusion gene and were considered atypical. However, the absence of cutaneous involvement, histologic features (spindle and a few epithelioid cells arranged in a nested pattern), and immunohistochemistry (diffuse positivity for S100 protein and HMB45) strongly suggested a diagnosis of CCS.

Therefore, we support the hypothesis that CCS belongs to the soft tissue sarcoma family and that the melanin synthesis is due to the specific action of the chimeric EWF/ATF1 protein via MITF activation. Molecular genetic abnormalities have been reported in 3 small series of CCS involving 10 patients, 12 patients, and 9 patients [3–5]. To our knowledge, the current study represents the largest reported series of patients with CCS with emphasis on mutational statue on BRAF, NRAS gene, and cytogenetic study of translocation (12, 22) (q13, q12).

Size constituted the only criterion that was useful as a predictor of the biologic behaviour of CCS (Table 3). In our series, on multivariate analysis, only tumor size (*P* : 0.01) emerged as a significant prognostic factor.

Tumor necrosis was also a significant indicator of poor prognosis, independent of tumor size [11, 12]. Indeed, we found that the presence of necrosis more than 50% associated with worse outcome. Of fourteen patients who had necrosis more than 50%, ten patients died, but on multivariate analysis, it was not a significant prognostic factor.

Unlike most soft tissue sarcomas, CCS often metastasizes to regional lymph nodes [1, 2]. Sixteen of our patients had lymph node metastases. A marked tendency for regional lymph node metastases has been observed by other authors, Chung and Enzinger reported a 53% rate, and Eckardt et al. [18] a 33% rate of lymph node metastases. Although we found lymph node metastases to be slightly less frequent than pulmonary metastases, they tended to occur earlier in the course of the disease. The role of regional lymph node dissection in CCS has not been established to date. Some authors recommend prophylactic elective lymph node dissection, whereas others suggest lymphadenectomy only in patients who show clinical lymphadenopathy [19]. Sentinel lymph node biopsy, which has been used successfully for the staging of patients with breast cancer and melanoma, may allow early detection of lymph node metastasis in patients with CCS [19, 20]. It is quite possible that, with diligent staging using sentinel node biopsy, some patients who are believed to have localized disease in the current series will turn out not to have localized disease, and this would have a significant impact on the analyses. Further investigations of the usefulness of sentinel lymph node biopsy in CCS are warranted.

Complete excision of the primary tumors appears to be the optimal approach to treatment, a conclusion that is shared by others [15]. Of the 29 patients who had local recurrences, 19 died of the tumors and 3 are alive with disease. These results suggest that local recurrence has a negative role in the management of CCS. In our series, successful treatment was best accomplished by excision with a wide surgical margin, with or without adjuvant radiation therapy. The presence of metastases, either at initial diagnosis or later in courses of the disease, was an ominous factor; no patients survived free of disease once metastatic CCS occurred.

The overall prognosis for CCS is poor [1–3]. In most cases, the disease pursues a relentlessly progressive course and terminates in death due to wide-spread dissemination. Although rapidly fatal progression may occur, late metastases are quite common after many years of freedom from disease. In our series, the 5-year survival was 59% at 5 years and 41% at 10 years. Similar findings have been reported in the literature (Table 3), with local recurrence or metastases (or both) occurring as late as 29 years after the initial surgery [2, 15]. Thus, once a diagnosis of CCS is made, the patient is at risk for recurrent disease for many years and must be followed closely. Awareness of delayed progression is especially important considering the young age of the most patients.

The rarity of the disease makes it difficult to draw conclusive statements, but clear cell sarcoma shares many features with melanoma, including histological, immunophenotypic, ultrastructural, and similarities in gene expression patterns. However, clear cell sarcoma is genetically distinct lacking melanoma-associated BRAF mutations and instead harboring recurrent and characteristic chromosomal translocations involving the EWSR1 gene. Although the term malignant melanoma of soft parts persist, it is important that this lesion be loosely considered a MM, but rather segregated as a unique tumor of soft tissue.

## Figures and Tables

**Table 1:** Clear cell sarcomas: clinical, histological, molecular, therapeutic, and follow-up data.

Case	Age	Sex	Site	Size (cm)	Treatment (*)	CHT regimens	Surgical margin	LRec (**)	LNMet (**)	Met (**)	FU (**)	Status	EWS- ATF1	Mitoses	Necrose	Grade	BRAF15	NRAS 61
1	36	F	Buttock	6	Surg+R+CT	IVA	pos	no	no	77	80	4	pos	10	0	2	neg	neg
2	23	F	Shoulder	9	CT+Surg+R	Adri	pos	no	no	9	12	4	pos	34	1	3	neg	neg
3	11	M	Heel	4	CT+Surg+R	I	neg	no	no	23	40	4	pos	12	1	3	neg	neg
4	53	F	Foot	6	CT+Surg+R	IVA	neg	14	no	7	16	1	pos	6	1	2	neg	neg
5	57	F	Forearm	5	Surg+R		neg	23	no	no	45	0	pos	5	0	2	neg	neg
6	23	M	Foot	2,5	Surg+R+CT	MAID; MTX, C	neg	no	no	no	11	0	pos	5	0	2	neg	neg
7	34	F	Forearm	4	Surg+CT+R	Adri,I, Cispl, F, Ditic	pos	1	0	0	8	4	pos	18	1	3	ni	ni
8	20	M	Hand	4	CT+surg+R	Adri, DTIC, limb perf	neg	no	no	no	28	0	pos	10	1	2	ni	ni
9	46	F	Hand	1,2	Surg		neg	24	no	no	40	0	pos	5	1	2	ni	ni
10	54	M	Foot	3	Surg+CT	MAID; MTX, C	pos	1	no	5	24	4	pos	18	2	3	ni	ni
11	45	F	Foot	3	Surg+R		pos	no	no	no	12	?	pos	8	1	2	ni	ni
12	48	M	Foot	1,8	Surg+R		pos	no	no	no	60	0	pos	3	2	3	neg	neg
13	37	M	Foot	2	Surg+R+CT	4 E+I, D	pos	no	no	57	120	1	pos	2	0	2	ni	ni
14	33	M	Leg	4	Surg+R+CT	6 I	neg	no	87	87	96	4	pos	10	2	2	ni	ni
15	31	M	Foot	?	Surg+CT+R	INF+Adri+T/EI	neg	no	0	10	16	4	pos	20	2	3	ni	ni
16	37	F	Foot	2	Surg+R+CT	Adri+I	pos	no	no	no	87	0	pos	9	0	2	neg	neg
17	42	F	Leg	6	Surg+CT+R	Adri+I	pos	no	54	no	60	1	neg	15	1	2	neg	neg
18	51	M	Thigh	15	Surg+R+CT	Adri+I+DTIC	pos	24	no	33	60	4	pos	12	2	3	ni	ni
19	9	M	Sacrum	7	Surg+R+CT	IVA	pos	12	no	12	20	4	ni	4	1	2	ni	ni
20	31	M	Knee	10	Surg+CT+R	I+C+V+DITC	neg	no	18	no	20	4	pos	2	1	2	neg	neg
21	45	M	Knee	4	Surg+CT+R	VACA	neg	no	no	no	48	0	ni	3	2	2	ni	ni
22	29	F	Foot	3	Surg+R		neg	12	no	16	40	4	pos	9	1	2	neg	neg
23	26	M	Foot	3	Surg+CT+R	Adri+I	neg	144	no	228	312	1	pos	5	1	2	neg	neg
24	38	F	Thigh	6	Surg+CT+R	VACA	pos	12	36	52	60	4	ni	9	1	2	ni	ni
25	17	M	Behind bladder	4	Surg+CT	Adri+I	neg	no	no	no	65	0	pos	10	1	2	ni	ni
26	6	F	Arm	8	Surg+CT	V+Carbo+ epirubicin	pos	1	0	no	180	0	ni	9	1	3	ni	ni
27	15	F	Scapula	5	Surg+CT	Carbo + Adria, IL2	pos	10	no	12	24	4	ni	2–4	1	2	ni	ni
28	36	M	Tibia	3	Surg+CT	Adri+I	pos	16	20	22	28	4	ni	10	1	2	ni	ni
29	15	F	Scapula	5	Surg+CT	IVA, IL2, DTIC	neg	no	no	no	12	0	ni	4	1	2	ni	ni
30	20	F	Scalp	4	Surg+CT+R	I+Adri	pos	84	72	96	120	4	neg	2	1	2	neg	neg
31	33	F	Hand	3	Surg+CT+R	Adri	pos	48	30	36	52	4	ni	10	1	2	ni	ni
32	32	M	Pelvis	9	Surg+CT+R	I+Adri	neg	6	10	21	32	4	ni	10	1	2	ni	ni
33	12	F	Foot	3	Surg+CT+R	IVA	pos	no	no	no	144	0	ni	2	1	3	ni	ni
34	14	M	Wrist	5 à 6	Surg+CT+R	IVA	neg	no	no	no	120	0	ni	10	1	2	ni	ni
35	29	M	Heel	2	Surg+CT+R	V+DITC+Adri +C	neg	24	no	76	82	4	ni	19	1	2	ni	ni
36	28	M	Wrist	3 à 4	Surg+CT+R	IVA, IL2, DTIC	pos	no	no	24	39	4	ni	11	1	2	ni	ni
37	10	M	Hip	5,5	Surg+CT+R	IVA	pos	68	72	no	120	0	pos	18	1	2	neg	neg
38	24	M	Foot	3	Surg+R		pos	no	no	no	156	0	ni	3	1	2	neg	neg
39	13	M	Leg	3	Surg+CT+R	VACA	neg	30	30	no	348	0	ni	21	1	2	ni	ni
40	47	F	Foot	3	Surg+CT+R	VACA	neg	24	no	no	240	1	ni	12	1	2	ni	ni
41	76	F	foot	4	Surg		pos	12	no	12	42	4	ni	2	1	3	ni	ni
42	34	M	Wrist	1	Surg+CT+R	VACA	neg	56	no	no	264	0	pos	10	1	2	neg	pos
43	40	M	Leg	4	Surg+CT+R	VACA	pos	60	no	84	96	4	neg	7	2	2	neg	neg
44	12	F	Foot	14	Surg+R		neg	36	no	no	84	0	ni	5	2	2	ni	ni
45	73	M	Scapula	10	Surg+CT+R	Adri+V+C+Plat	pos	48	36	24	96	4	ni	29	2	3	ni	ni
46	20	M	Scapula	6	CT+Surg+R	Adri+V+ DTIC+C	pos	18	18	18	24	4	pos	8	1	2	neg	neg
47	33	M	Foot	5	Surg+CT	F	pos	16	4	4	31	4	pos	9	1	2	neg	neg
48	81	F	Foot	3	Surg		pos	240	no	250	254	4	ni	9	2	2	ni	ni
49	46	M	Foot	4	Surg+CT+R	I	neg	60	72	250	260	4	pos	24	2	3	pos	neg
50	47	M	Hand	6	Surg+CT+R	VACA	neg	no	no	96	144	4	pos	15	2	2	neg	neg
51	29	F	Foot	2	Surg		pos	no	60	no	60	1	ni	10	2	2	ni	ni
52	30	M	knee	10	Surg+CT+R	C+V+ Adri+DTIC	pos	no	6	18	36	4	pos	5	2	2	neg	neg

((*) Surg: surgery, R: radiotherapy, CT: chemotherapy. (**) LRec: local recurrence (months), LNMet: locoregional lymph node metastasis (months), Met: distant metastasis (months), and FU: followup (months). Status: 0: alive with no disease, 1: alive with disease, and 4: died of disease. Mitoses: number of mitoses per ten high power fields. neg: negative, pos: positive, ni: not identified. Necrosis: 0: no necrosis, 1: less than 50% of necrosis, 2: more than 50% of necrosis). A: actinomycin D; Adri: Adriblastina, C: cyclophosphamide, Carbo: carboplatin, cisplt: cisplatin; DITIC: dacarbazine, EPI: eoposide, F: fotemustine; I: ifosfamide, IL2: Interleukin 2, IVA: ifosfamide (3 g/m²) + dactinomycin (1,5 mg/m²) + vincristine (1,5 mg/m²); Limb perf: regional isolated limb perfusion with melphalan and tumor necrosis factor alpha, MTX: methotrexate, MAID: doxorubicin (15 mg/m²) + dacarbazine (250 mg/m²/j) + ifosfamide (2–2,85 g/m²/j), V: vincristine; VACA: cyclophosphamide (1,2 g/m²) + doxorubicin (30 mg/m²) + dactinomycin (0,5 mg/m²) + vincristine 1,5 mg/m²; INF: intrferon; T: taxol.

**Table 2:** Multivariate analysis of overall survival.

Variable	Number	5-year survival univariate *P *	Event-free Survival
Sex			
Men	30	0,83	0,70
Women	22		
Age			
≤30 y	22	0,32	0,38
>30 y	30		
Size			
≤4 cm	30	0,06	0,01
>4 cm	21		
Mitoses			
<10	27	0,61	0,44
≥10	25		
Necrosis			
<50%	38	0,66	0,47
≥50%	14		
Grade			
2	40	0,19	0,16
3	12		

**Table 3:** Clinicopathologic studies of clear cell sarcoma.

Reference	Year	No. of patients	Five-year survival	Prognostic factors
Sara et al. [9]	1990	17	40	Size
El-Naggar et al. [10]	1991	11	NA	DNA content
Lucas et al. [11]	1992	35	67	Size, necrosis
Montgomery et al. [12]	1993	58	63	Size*, necrosis*
Deenik et al. [13]	1999	30	30	Size, radiotherapy
Finley et al. [14]	2001	8	55	Size
Ferrari et al. [15]	2002	28	66	Size, site, IRS group
Takahira et al. [16]	2004	14	33	Mitosis
Kawai et al. [17]	2007	75	47	Size,* depth, sex, TNM
Current study	2005	52	59	stage, surgical margin size*

NA indicates not available; IRS group, Intergroup Rhabdomyosarcoma Study Group.

*Multivariate analysis.
